# Multiband Antennas for Modern Smartphones: A Review from Sub-1 GHz to Millimeter-Wave and Toward 6G

**DOI:** 10.3390/s26165156

**Published:** 2026-08-14

**Authors:** Yiming Fan, Rongrong Dong, Yuming Wu, Changjiang Deng

**Affiliations:** 1 Key Laboratory of Ministry of Industry and Information Technology, State Key Laboratory of Environment Characteristics and Effects for Near-Space, School of Integrated Circuits and Electronics, Beijing Institute of Technology, Beijing 100081, China; 2 Zhuhai Key Laboratory of Terahertz Intelligent Sensing and Wireless Communication Systems, Terahertz Science Application Center (TSAC), Beijing Institute of Technology, Zhuhai 519088, China

**Keywords:** multiband smartphone antennas, mobile terminal antennas, multi-antenna systems, millimeter-wave antennas, Sub-6 GHz/millimeter-wave integration, reconfigurable antennas

## Abstract

The rapid development of wireless communication systems has increased the complexity of smartphone antenna design. Modern terminals are required to simultaneously support Sub-1 GHz cellular bands, 5G New Radio (NR), millimeter-wave communication, satellite links, and emerging sensing services within limited physical space. As a result, multiband operation has become a central challenge for mobile terminals. This paper reviews the recent research advances in multiband smartphone antennas, organized according to the evolution from single-antenna to multi-antenna architectures and including both Sub-6 GHz and millimeter-wave applications. Single-antenna Sub-3 GHz techniques, multiband multi-antenna systems, dual-band millimeter-wave antennas, and integrated Sub-6 GHz/millimeter-wave architectures are systematically summarized. The key technologies, including multi-mode cooperation, characteristic-mode design, multiple-input multiple-output (MIMO) decoupling, and shared-aperture integration, are discussed. The emerging B5G/6G-oriented technologies are also highlighted. This review provides an overview of current design strategies and future trends for next-generation multiband smartphone antennas.

## 1. Introduction

### 1.1. Evolution of Smartphone Antennas

**Figure 1:**
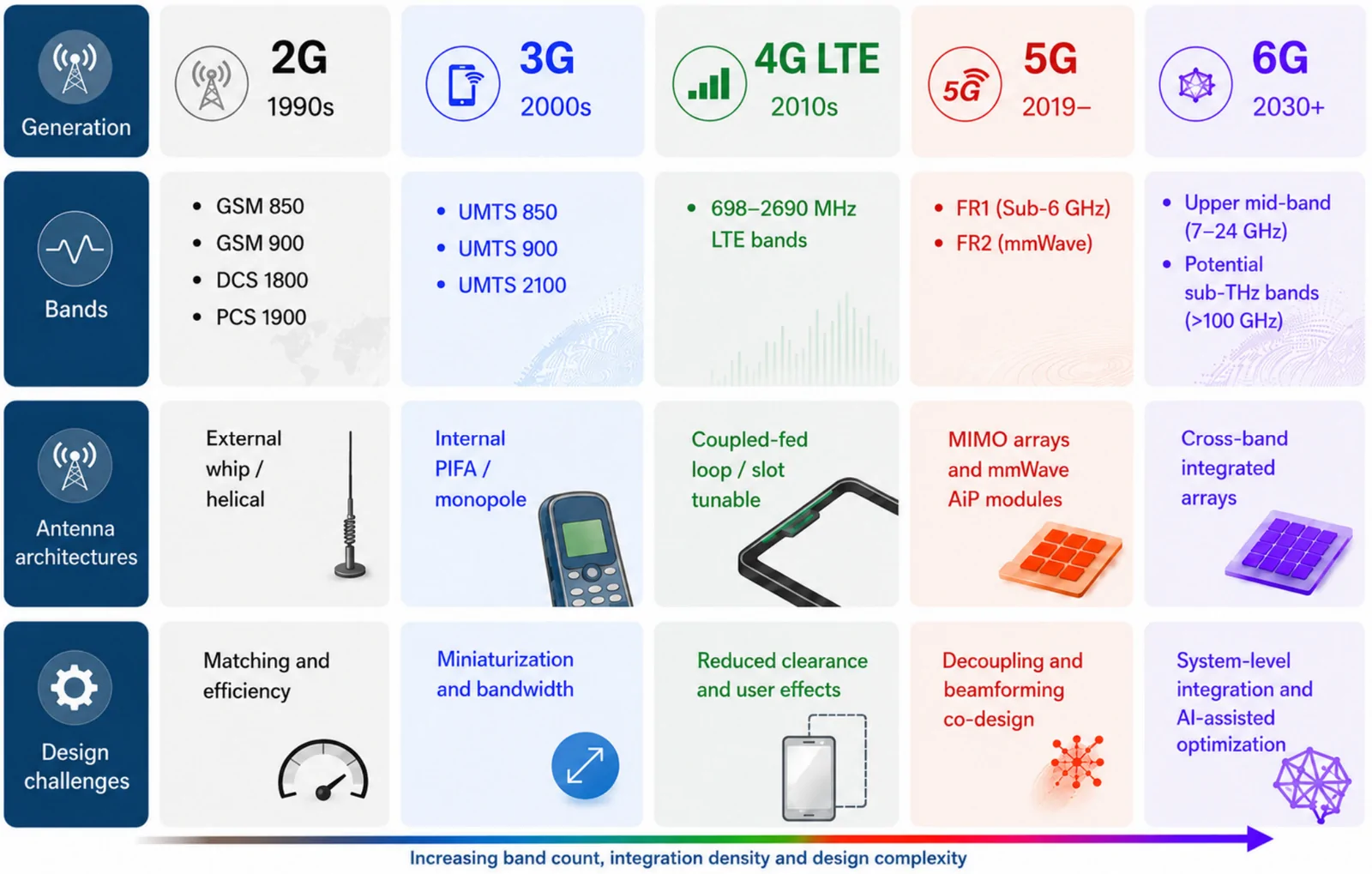
Evolution of smartphone antenna bands, architectures, and design challenges from 2G to prospective 6G systems.

The rapid evolution of wireless communication systems has profoundly transformed smartphone antenna design. Early mobile terminals were designed primarily for narrowband GSM services. Modern smartphones, however, must support a much broader spectrum covering 2G (generation), 3G, 4G LTE, 5G Sub-6 GHz, and millimeter-wave communications [1,2]. In addition, future 6G systems are expected to extend toward the candidate upper-mid-band and Sub-terahertz (THz) spectrum [3,4]. This continued spectrum expansion makes multiband smartphone antenna design an increasingly important topic which needs further investigation in mobile terminal engineering [5]. The evolution of smartphone antenna bands, architectures, and design challenges across wireless generations is summarized in Figure 1.

In the 2G era, handsets were mainly designed for one or two narrow bands, like GSM850 (824–894 MHz) and GSM900 (880–960 MHz). External whip and helical antennas were commonly used because of their simple implementation and acceptable radiation efficiency [5]. With the arrival of 3G, additional bands such as DCS1800 (1710–1880 MHz), PCS1900 (1850–1990 MHz), and WCDMA2100 (1920–2170 MHz) were introduced. Smartphone antenna designs therefore shifted toward integrated and low-profile structures. Typical designs included planar inverted-F antennas (PIFAs), printed monopoles, and loop antennas, which provided compact configurations and multiple resonant modes [6,7].

With the evolution of mobile communications, the transition to 4G LTE marked a major turning point in smartphone antenna design. LTE introduced a highly fragmented global spectrum and required a single device to cover the frequencies from 698 MHz to 2690 MHz [8,9]. As a result, antenna designers had to address challenges including low-frequency miniaturization, multi-resonance generation, and wideband impedance matching within increasingly limited ground-clearance regions. A wide range of techniques was therefore investigated, including coupled feeding [10], parasitic branches [11], capacitive coupling elements [12], embedded parallel resonant structures [13], and frequency-reconfigurable architectures using PIN diodes, RF-MEMS, or digitally tunable capacitors [14,15]. Meanwhile, the growing popularity of metal-rimmed industrial designs further promoted bezel-integrated antennas, in which the metallic frame contributes directly to the radiation [16].

The commercialization of 5G further increased the complexity of smartphone antenna design. Modern terminals must support both Sub-6 GHz and millimeter-wave communication systems within the same compact platform [17]. For Sub-6 GHz operation, antennas are required to cover the new 5G NR bands, including n41 (2.496–2.690 GHz), n77 (3.3–4.2 GHz), n78 (3.3–3.8 GHz), and n79 (4.4–5.0 GHz), while maintaining compatibility with existing 4G services. These requirements are usually combined with multiple-input multiple-output (MIMO) operation, which demands high isolation among multiple antennas placed in limited bezel regions [18,19]. For millimeter-wave operation, typically in the 24–40 GHz range, the severe propagation loss requires highly directional phased arrays with beam-steering capability. Metal-frame-integrated arrays [20] and antenna-in-package (AiP) solutions [21] have therefore become important implementation approaches. The simultaneous integration of Sub-6 GHz antennas and millimeter-wave modules leads to new challenges such as cross-band coupling, spatial reuse, and electromagnetic co-design [22,23].

In summary, smartphone antennas have evolved from narrowband radiators to compact multiband antenna systems. This evolution imposes increasingly stringent requirements on antenna volume, spectrum coverage, port counts, and robustness to user effects. Existing reviews generally focus on the overall evolution of handset antennas or on specific topics such as 5G MIMO, decoupling, and millimeter-wave arrays. In contrast, this review organizes the literature around multiband operation across different frequency ranges and levels of system integration. In the following sections, representative techniques are systematically classified and compared to clarify their development and integration in modern smartphones.

### 1.2. Motivation and Scope of This Review

**Figure 2:**
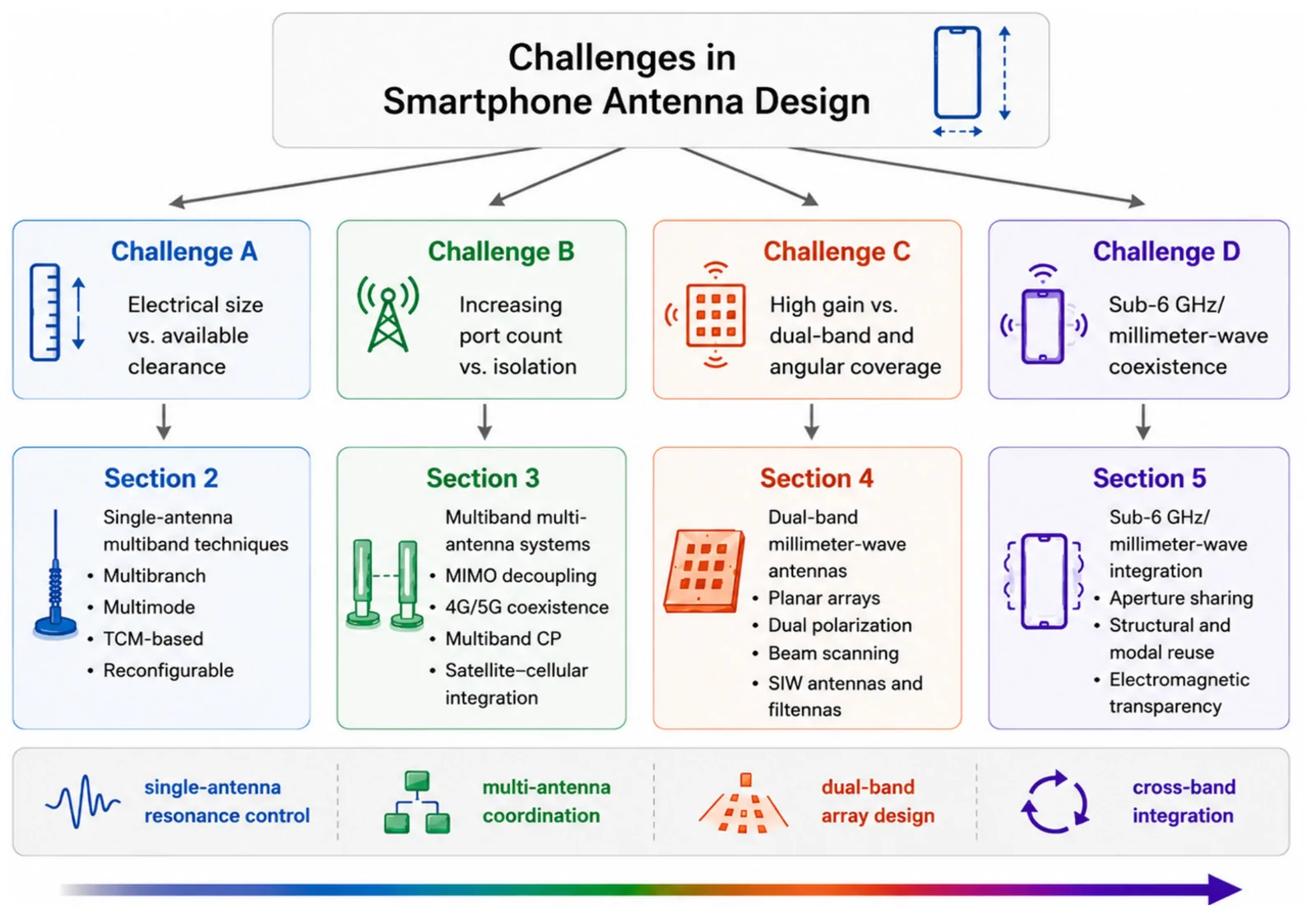
Main design challenges in multiband smartphone antennas and their correspondence to Section 2, Section 3, Section 4 and Section 5.

Multiband smartphone antenna design faces increasingly stringent constraints with the evolution of wireless generations. This section specifies the essential interrelated design aspects that influence multiband smartphone antenna performance. Their relationships with the corresponding technical sections are summarized in Figure 2 and discussed in detail in Section 2, Section 3, Section 4 and Section 5.

A. The fundamental physical limits of electrically small antennas impose a strong tradeoff among antenna size, bandwidth, and radiation efficiency [24,25]. Meanwhile, the pursuit of higher screen-to-body ratios has continuously reduced the available ground clearance in modern handsets. For example, the recently reported metal-bezel designs can provide clearances even below 2 mm [16]. In addition, cellular frequency allocations have continued to extend toward lower frequencies. Therefore, the growing mismatch between the required electrical size and the available physical space creates a significant challenge in single-antenna multiband design. Extensive research on the solutions to overcome this challenge is reviewed in Section 2 in terms of multi-branch structures, multi-mode operation, characteristic-mode-based design, and frequency-reconfigurable techniques.

B. Modern handsets must support multiple 4G LTE and 5G NR bands and functions within the same narrow chassis. When one radiator is shared by several closely spaced bands, such as n41, n78, and n79, the cross-band coupling becomes a major research bottleneck. This challenge cannot be effectively addressed by conventional same-band decoupling techniques. Therefore, filter-loaded and self-decoupled antenna-pair designs have been proposed [26,27]. In addition, 4G and 5G networks will coexist for a long time. This requires different radiators to reuse the same limited clearance through compact branch sharing and physical integration [28]. Moreover, direct-to-cell satellite services introduce new requirements for multiband endfire circular polarization performance for handsets [29,30]. Section 3 summarizes such studies of multiband multi-antenna systems, including MIMO antenna decoupling, 4G/5G coexistence layouts, and circularly polarized (CP) satellite–cellular antennas.

C. The tradeoff between millimeter-wave high-gain demand and multiband coverage should be taken into account. In fact, millimeter-wave operation introduces different design constraints from those of Sub-6 GHz antennas. Millimeter-wave signals suffer from much higher free-space path loss and are more sensitive to blockage and diffraction effects [31]. For example, according to the Friis transmission formula, the free-space path loss at 28 GHz is about 20.6 dB higher than that at 2.6 GHz with the same propagation distance. Furthermore, the fragmented global allocation of the millimeter-wave spectrum covering 24 to 30 GHz and 37 to 43.5 GHz makes it preferable for a single terminal to cover two or more discrete millimeter-wave bands, which is implemented by the same shared aperture rather than several individual single-band modules [3,32,33]. Section 4 reviews compact dual-band millimeter-wave antennas with high gain, beam-scanning capability, polarization diversity, and inter-band isolation, including multi-mode microstrip and dipole arrays, dual-polarized structures, and substrate-integrated waveguide (SIW)-based antennas.

D. Since Sub-6 GHz and millimeter-wave coexist on a single platform, a distinct challenge of modern 5G smartphones is the careful arrangement of corresponding systems within the same compact platform. These two antenna systems differ greatly in their respective operating wavelength, radiation mechanism, and physical size. In portable smartphones, they are often placed in close proximity. The resulting near-field coupling and capacitive loading lead to the key issue of cross-band integration [22,23]. Recent studies have mainly suggested the solution in three ways. The first is to share a common aperture or physical surface. The second is to reuse one metallic structure to excite different modes at different frequencies. The third is to introduce electromagnetic-transparency, band-stop, or decoupling mechanisms to suppress the mutual coupling between the two systems. These strategies are analyzed in Section 5.

Based on the current designs, Section 6 discusses emerging B5G and 6G directions, including integrated sensing and communications (ISAC)-oriented cross-band integration, AI-assisted antenna design and evaluation, and operation in the FR3 and Sub-THz ranges. Finally, Section 7 concludes the review and outlines the remaining research challenges.

## 2. Multiband Single-Antenna Techniques

To accommodate the rapidly increasing number of operating bands within the limited space of modern handsets, a common strategy is to enable a single antenna structure to support multiband or wideband operation. Existing studies can be broadly classified into three design approaches. The first approach introduces additional radiating branches or parasitic elements with different electrical lengths to generate multiple resonances. The second approach exploits the inherent eigenmodes of metallic structures already existing in the terminal, such as the chassis or metal bezel, through characteristic-mode-guided excitation. The third approach employs active components, such as PIN diodes, varactors, and RF switches, to dynamically reconfigure the antenna states and a single compact aperture to support multiple bands. The following three subsections review representative works based on these approaches.

### 2.1. Multi-Branch and Multi-Mode Cooperation

Adding physical radiating structures is the most direct approach to support single-antenna multiband operation. This approach can be further divided into multi-branch and multi-mode designs. In multi-branch designs, different radiating branches are assigned to different operating bands. Each branch is typically tuned to produce one or two resonant frequencies. In multi-mode designs, multiple resonant modes of a shared radiating structure are deliberately excited and merged to form a broader operating band.

**Figure 3:**
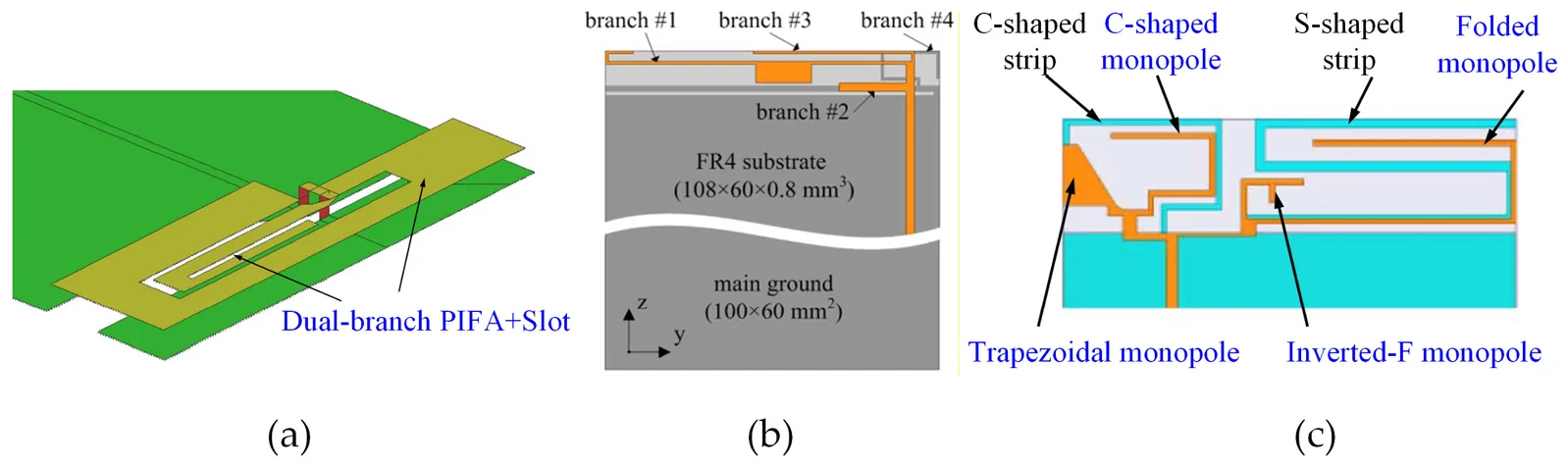
Multi-branch antenna structures. (**a**) Dual-branch PIFA with a slot [6]. (**b**) Multiple branches, open slot, and tuning pad [35]. (**c**) Folded, C-shaped, and trapezoidal monopole branches [36].

Multi-branch with independent resonances: The multi-branch technique drives multiple radiating branches of distinct electrical lengths from a single feed port so that each branch resonates individually at its target band [6,8,9,13,34,35,36]. As shown in Figure 3a, a PIFA and a slot radiator were combined with a single feed [6]. The two radiators operate independently at different frequency bands, enabling coverage of GSM900/1800/1900, UMTS, and S-DMB. A representative extended design is illustrated in Figure 3b, where multiple monopole branches are combined with a slotted ground to offer distinct resonances, like the 0.25λ and 0.75λ modes, within the desired bands [35]. A common refinement is to add parasitic branches near the driven elements, so that the additional resonances are generated through the near-field coupling [36]. As illustrated in Figure 3c, each folded, C-shaped, and trapezoidal monopole branch is coupled to a parasitic strip to operate at 900 MHz, 2 GHz, and 3.5/5 GHz bands. These designs mainly rely on the branch-level resonance allocation, rather than the mode merging on the shared radiating structure.

Multi-mode cooperation on a shared radiator: Mode-merging strategies have been widely adopted in compact and metal-rimmed handset antennas [37,38,39,40,41]. These methods focus on multiple resonant modes, such as different loop orders or coupling-induced auxiliary modes, on a common radiator. Figure 4 in [37] reports a compact loop antenna supporting six resonant modes within a single low-profile structure. A dual-functional parasitic element is excited as both an unbalanced 0.25λ mode and a balanced 0.5λ mode, and these two parasitic modes are then merged with the inherent higher-order modes of the primary loop, forming continuous coverage from 660 to 1100 MHz. Compared with the multi-branch designs, this approach does not increase the number of physical radiators. Instead, it increases the number of useful modes excited and matched on each radiator. This transition from adding resonators to engineering modes on a shared structure naturally leads to the characteristic-mode-based designs (which will be discussed in Section 2.2), where the shared radiator is no longer a custom-shaped branch but an existing metallic part of the terminal, like the chassis or metal bezel.

### 2.2. Characteristic-Mode-Based Design

**Figure 5:**
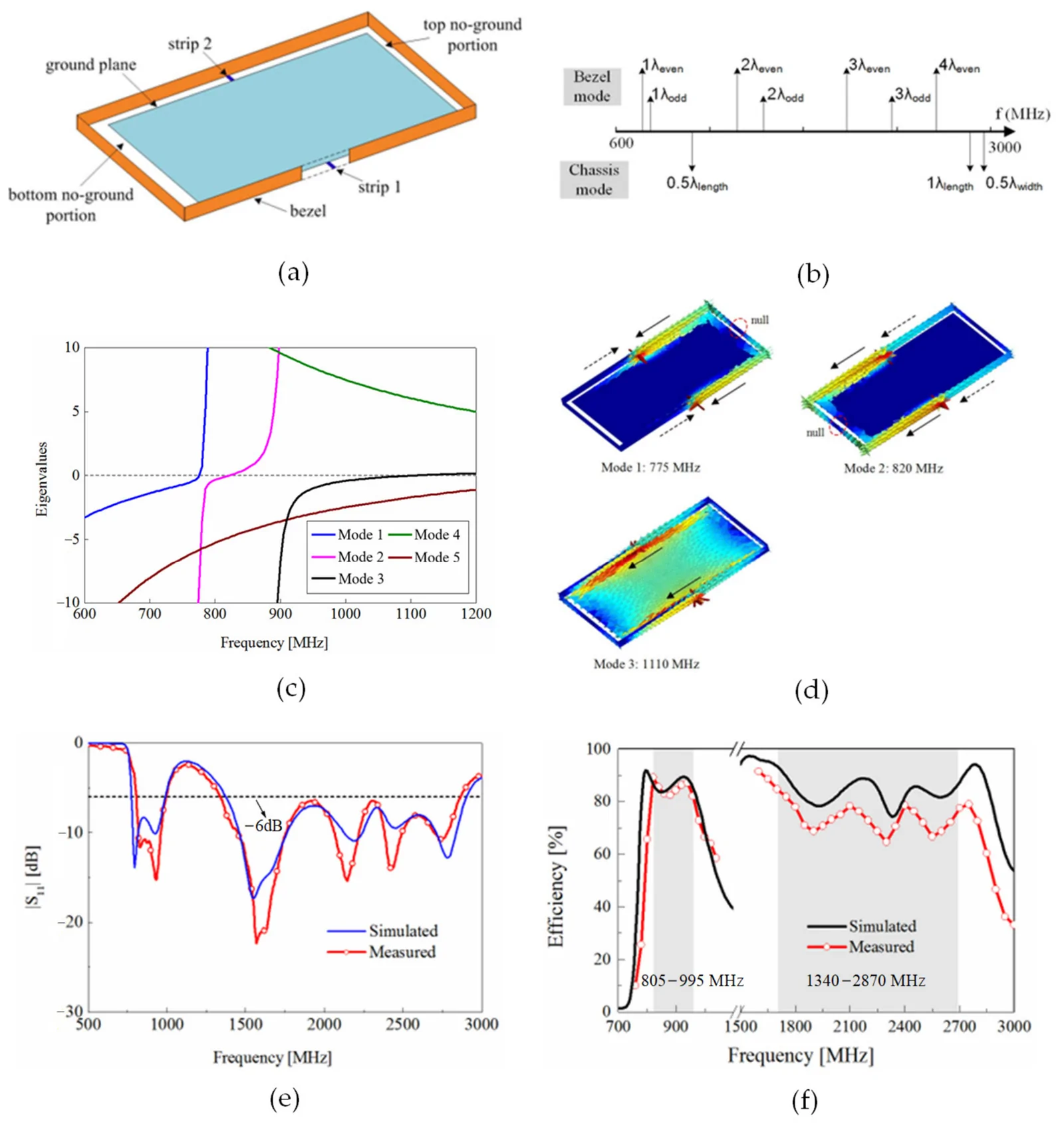
TCM-based unbroken metal bezel antenna [16]. (**a**) Antenna structure. (**b**) Modal significance. (**c**) Eigenvalues of the bezel model in the lower band. (**d**) Normalized eigencurrents of the bezel model. (**e**) Simulated and measured S11. (**f**) Simulated and measured total efficiency.

In clearance-limited smartphone platforms, the theory of characteristic modes (TCM) provides an excitation-independent design method for analyzing and reusing the inherent modal properties of terminal metallic structures [16,42,43,44,45]. A representative metal-frame antenna architecture is reported in [16], as illustrated in Figure 5. Unlike the conventional wideband resonant structures, this design analyzes the characteristic modes of an unbroken metal bezel. Wide multiband coverage is achieved using a single feed. Meanwhile, the structural integrity of the metal frame is preserved. For Sub-1 GHz operation, the feed point and connecting strips are optimized to excite and merge the 0.5λ even and odd modes of the bezel, enabling coverage from 805 to 995 MHz. For the higher Sub-3 GHz band, a 7-nH lumped inductor is introduced to assist the matching of higher-order modes. This allows the antenna to further cover 1340–2870 MHz. With a compact feeding and tuning structure, the eigenmodes of the terminal can be selectively excited and combined. This reduces the need for additional radiating branches and improves spatial utilization in smartphone antenna systems.

### 2.3. Frequency-Reconfigurable Techniques

**Figure 6:**
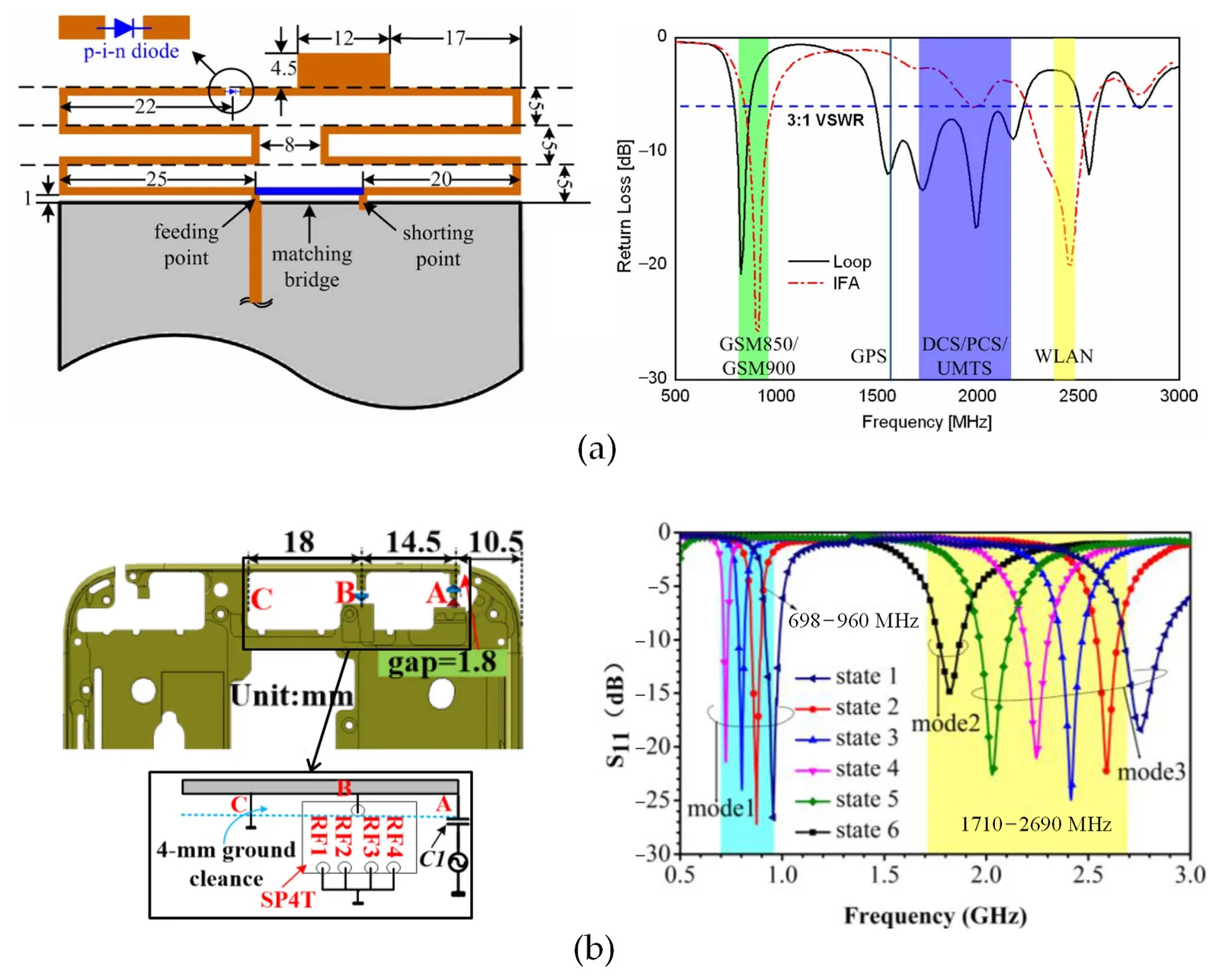
Frequency-reconfigurable smartphone antennas using active components. (**a**) Metal-frame antenna loaded with PIN diodes for multiband operation [46]. (**b**) Metal-frame slot antenna loaded with an SP4T RF switch for tunable LTE coverage [47].

Frequency-reconfigurable techniques are another effective method for covering multiple cellular bands within a compact antenna volume [46,47,48,49,50]. By using active components, such as PIN diodes, varactor diodes, or RF switches, the antenna can be switched among different operating states. In this way, one compact radiator can cover several frequency bands without increasing any antenna footprint. Figure 6a illustrates a folded loop-IFA antenna [46]. By controlling a single PIN diode, the antenna can switch between a closed-loop mode and an open inverted-F mode. As a result, the antenna covers the GSM850/900, GPS, DCS, PCS, UMTS, and WLAN bands within a compact volume of 60 × 5 × 5 mm^3^. In ref. [47], an SP4T RF switch was used to load different lumped reactances on a metal-frame loop antenna, as shown in Figure 6b. The antenna covers 698–960 and 1710–2690 MHz within an area of 32.5 × 4 mm^2^. The reconfigurable approach is promising for practical smartphone antennas because it improves frequency coverage without requiring additional radiating branches. However, the active components may introduce unwanted insertion loss and reduce the radiation efficiency.

### 2.4. Summary and Comparison

Table 1 summarizes representative single-antenna multiband techniques. Multi-branch designs enable relatively independent band allocation, whereas multi-mode and TCM-based approaches exploit multiple resonances or characteristic modes to improve spectral and structural utilization. Reconfigurable antennas extend the achievable frequency coverage within a compact footprint but introduce switch loss, parasitic effects, and biasing requirements.

## 3. Multiband Multi-Antenna Systems

Modern handsets increasingly rely on multiple radiating elements to support a rapidly expanding spectrum. This trend has introduced new challenges such as undesirable mutual coupling, reduced radiation efficiency, and multiband coexistence coordination. This section analyzes four typical research directions. The first is dual- and tri-band antenna pairs, where the decoupling strategy has evolved from same-band isolation to cross-band suppression. The second is 4G and 5G coexistence layout, where different radiators are physically reused within extremely limited space. The third is multiband CP antennas for direct-to-cell satellite links. Finally, integrated satellite and cellular antennas are reviewed.

### 3.1. Multiband MIMO Antennas

**Figure 7:**
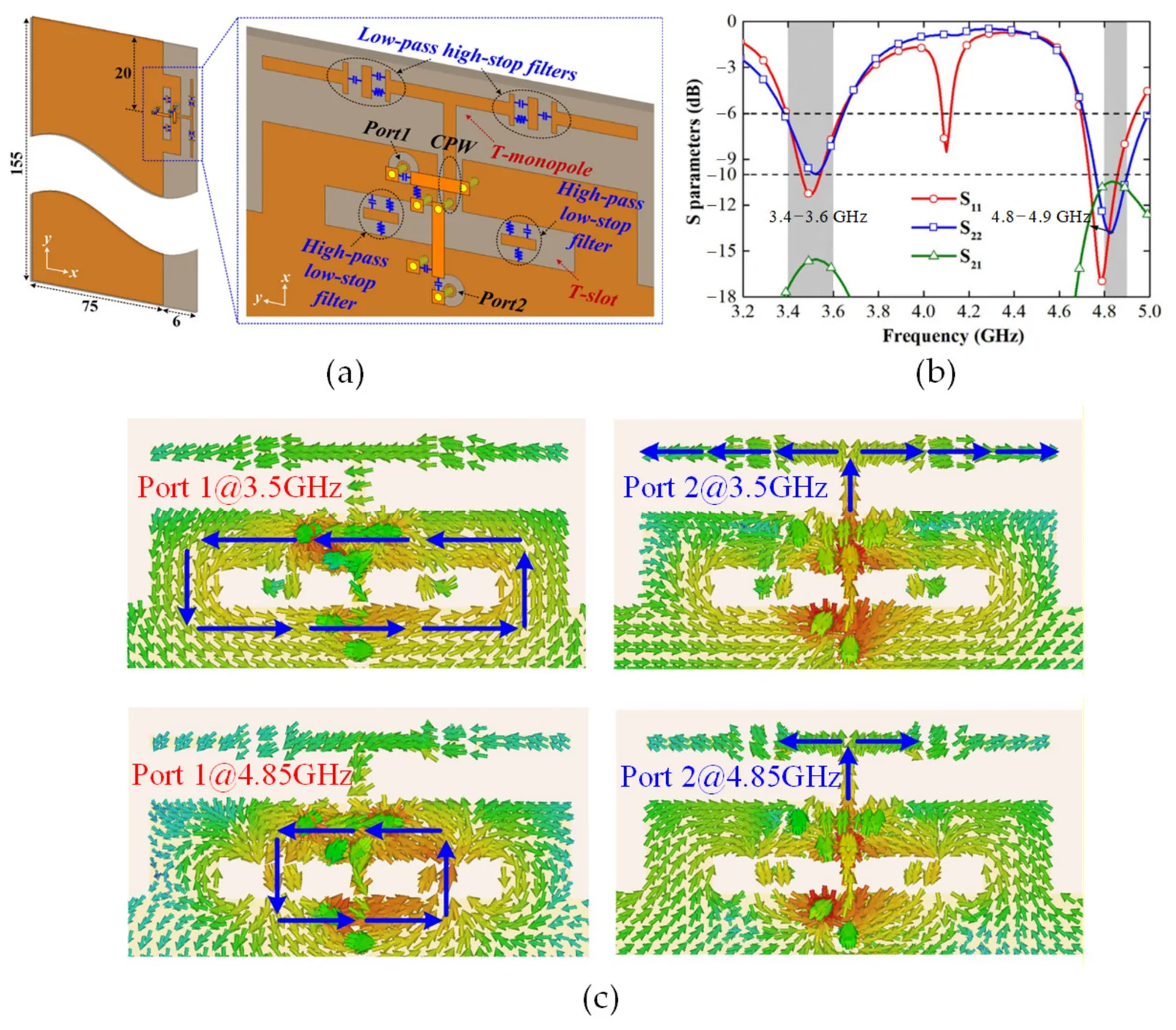
Dual-band decoupling antenna pair with integrated low-pass and high-pass filtering branches [26]. (**a**) Antenna configuration. (**b**) Simulated S-parameters. (**c**) Vector current distributions at different operating frequencies.

**Figure 8:**
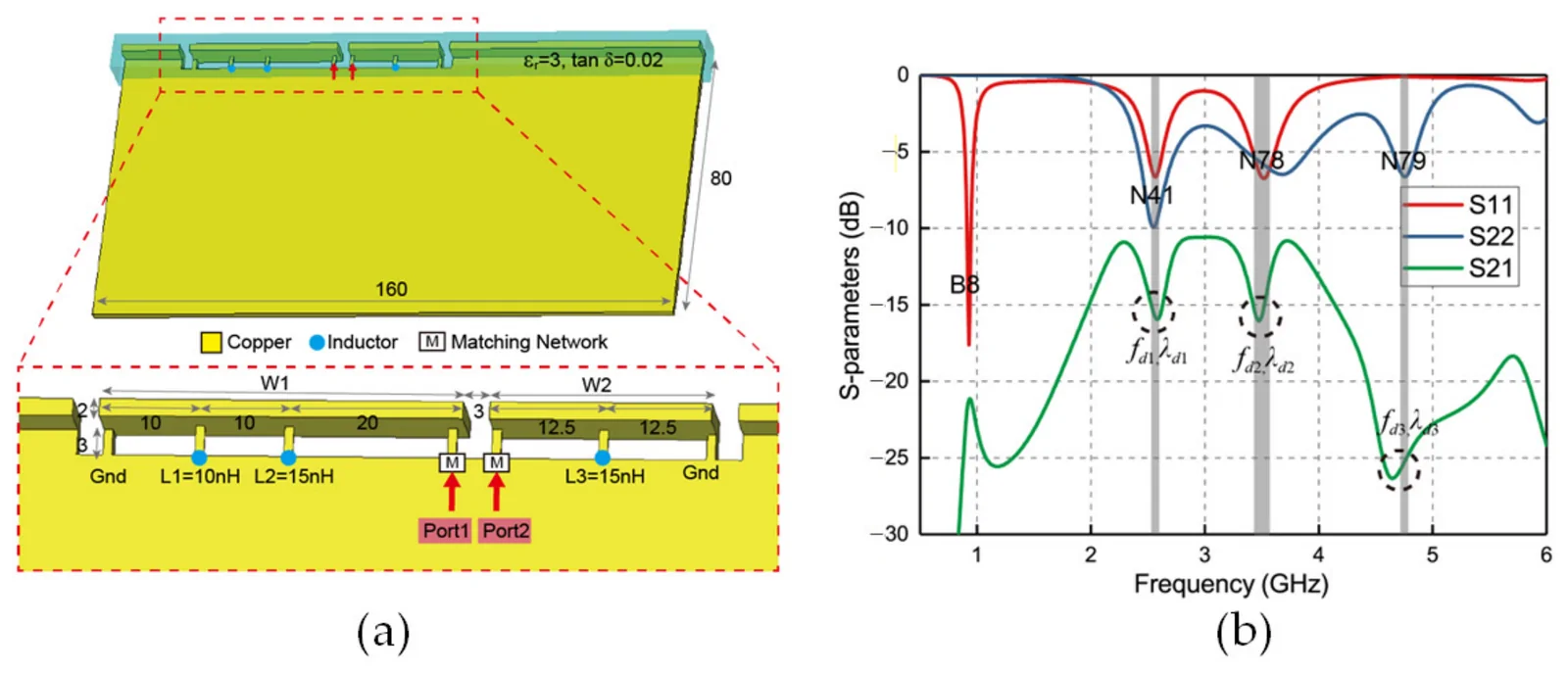
Self-multipath decoupling antenna pair supporting three 5G NR bands for smartphone applications [53]. (**a**) Antenna configuration. (**b**) Simulated S−parameters.

Dual-band 5G MIMO systems often cover closely spaced bands such as n78 and n79. In compact layouts, cross-band mutual coupling suppression becomes a major difficulty. Several recent works have addressed this issue by using the filter-loaded or self-decoupled antenna pairs [26,27,51,52]. In ref. [26], a lumped-filter-based decoupling architecture was proposed, as shown in Figure 7. Both low-pass and high-pass filtering branches were integrated into the compact orthogonal T-shaped radiating branches. The frequency-dependent impedances of the lumped elements introduce an additional resonant mode and suppresses coupling between the low- and high-frequency channels. In ref. [53], an asymmetric self-multipath decoupling antenna pair was developed to support isolation enhancement across three 5G NR bands, including n41, n78, and n79, as illustrated in Figure 8. Separate coupling nulls are generated in each band through joint tuning of the two asymmetric antenna elements.

### 3.2. 4G/5G Coexistence Architecture

**Figure 9:**
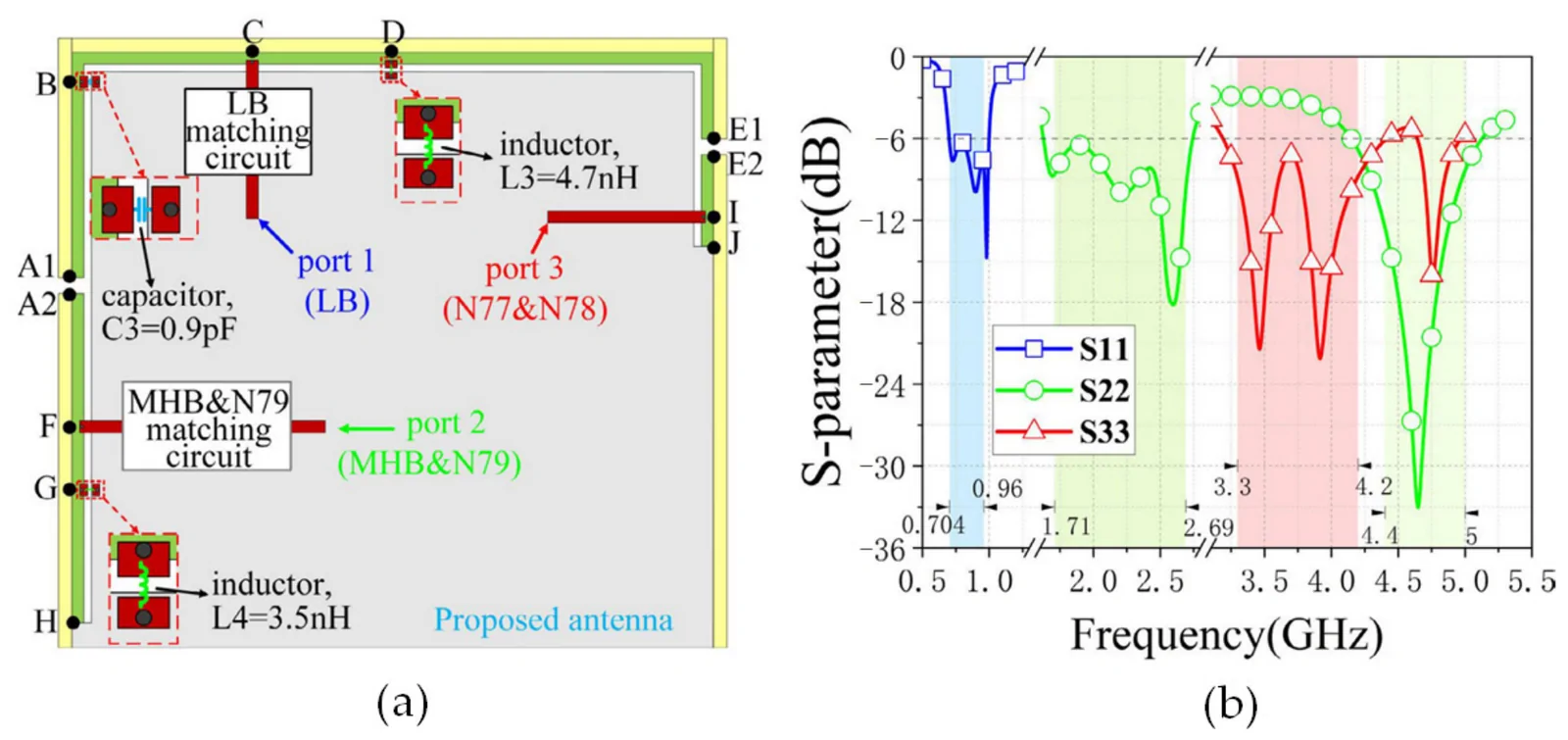
Seamless 4G/5G coexistence through antenna branch reuse [28]. (**a**) Antenna configuration. (**b**) Simulated S-parameters.

4G and 5G networks will coexist for a relatively long period. As a result, modern smartphones are required to retain complex Sub-3 GHz antenna systems and integrate additional Sub-6 GHz 5G multi-antenna modules as well. Consequently, the narrow spacing between the metal bezel and the screen poses challenges for cross-band reuse and small-clearance antenna design [28,54,55,56]. In ref. [28], an eleven-band shared-branch mobile antenna with a 0.5 mm ground clearance was developed, as shown in Figure 9. It is noted that the lumped capacitors are introduced to enable the low-band antenna branches to be reused by both the middle-and high-frequency band and 5G NR antennas. This design achieves broad coverage of 4G LTE and 5G NR bands on a compact metal-bezel platform.

### 3.3. Multiband Circularly Polarized Antennas

**Figure 10:**
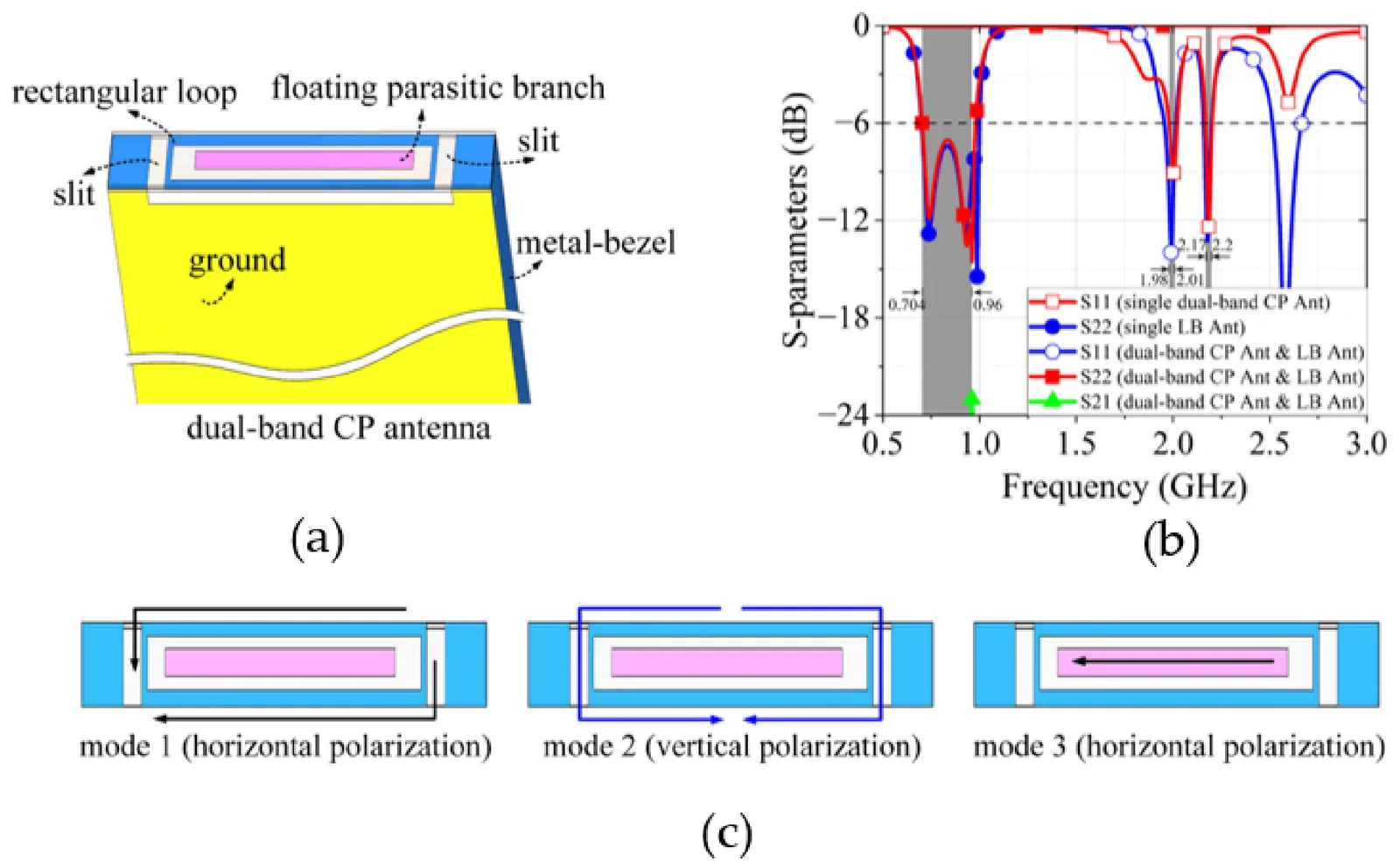
Dual-band endfire circularly polarized antenna [29]. (**a**) Design concept of the dual-band CP antenna. (**b**) Simulated S-parameters. (**c**) Operating modes of the dual-band CP antenna.

**Figure 11:**
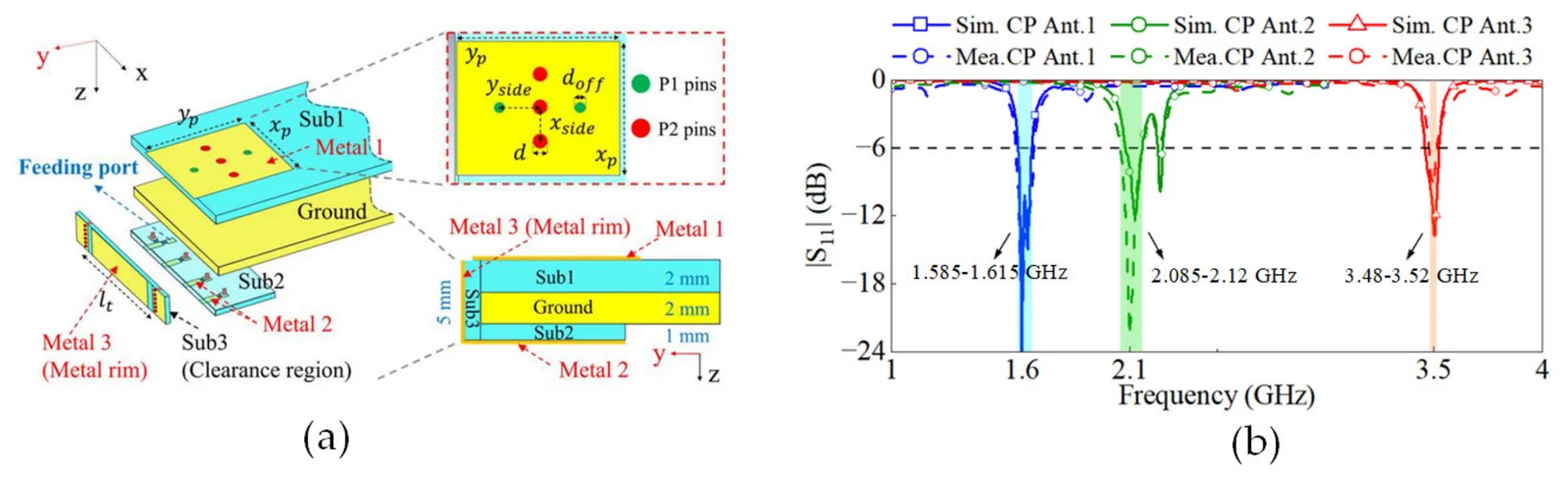
Tri-band reconfigurable endfire circularly polarized antenna [57]. (**a**) Antenna configuration. (**b**) Simulated S-parameters.

Direct phone-to-satellite communication has stimulated research into multiband endfire CP antennas for mobile terminals [29,57]. CP radiation is advantageous for satellite links because it can mitigate the Faraday rotation effect. For the n256 (1.98–2.01 GHz UL and 2.17–2.20 GHz DL) direct-to-cell band, a dual-band endfire CP antenna was proposed in [29], as shown in Figure 10. A folded rectangular loop and a floating parasitic branch excite two orthogonal polarization modes. Their combination generates endfire CP radiation along the metal bezel. The antenna can also coexist with the conventional cellular antennas. In ref. [57], a tri-band frequency-reconfigurable endfire CP antenna was reported, as illustrated in Figure 11. With a single feeding port, the intact top metal serves as the horizontally polarized radiator, and switchable pins are loaded on a vertically polarized parasitic patch. By changing the switching states, the antenna achieves CP operation at 1.6, 2.1, and 3.5 GHz.

### 3.4. Integrated Satellite and Cellular Antennas

**Figure 12:**
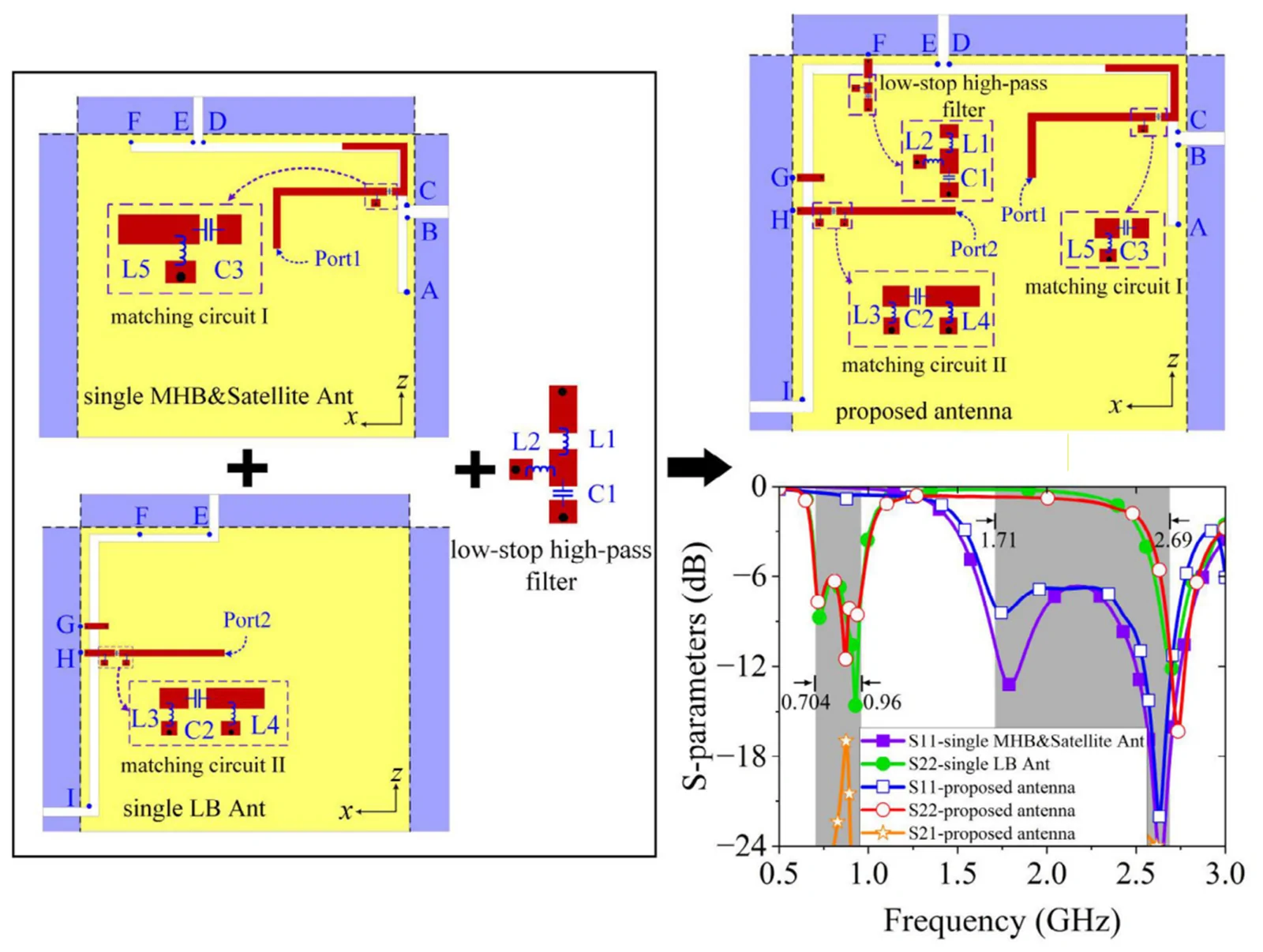
Co-design of a wide-beamwidth endfire satellite antenna and a 4G LTE cellular antenna [58].

To support both the terrestrial and non-terrestrial networks in the same device, cellular and satellite antennas must coexist within a compact metal-bezel smartphone [29,30,58]. In ref. [58], an octa-band mobile antenna was proposed for n256 direct-to-cell satellite communication and Sub-6 GHz cellular operation, as shown in Figure 12. The antenna provides a wide beamwidth in the endfire direction for satellite links. A 0.5λ floating branch is excited to generate the endfire radiation, while two parasitic branches are coupled to extend the middle- and high-frequency cellular bands. Moreover, a low-stop and high-pass filter is used to enable branch reuse between the satellite and cellular antennas. This design supports both LTE/5G terrestrial bands and the n256 satellite band on a compact metal-bezel platform.

### 3.5. Summary and Comparison

Table 2 summarizes representative multiband multi-antenna systems. These designs employ polarization or modal orthogonality, self-decoupling, branch reuse, and satellite–cellular integration to support multiple bands within limited space. Increasing the number of bands and ports generally makes current-path control, matching, isolation, and polarization tuning more involved.

## 4. Dual-Band Millimeter-Wave Antennas

To satisfy the increasing demand for high-data-rate wireless services, the millimeter-wave spectrum has become an indispensable component of 5G mobile communications. Typical smartphone millimeter-wave bands are mainly distributed in the 24–30 and 37–43.5 GHz ranges. Although these bands provide considerable spectrum resources, they suffer from severe free-space path loss due to the millimeter-wave signals. Another practical challenge is that millimeter-wave frequency allocations differ across various regions. To ensure global compatibility, a single smartphone is expected to support multiple discrete millimeter-wave bands. This demand has motivated extensive research on compact dual-band millimeter-wave antennas and arrays.

### 4.1. Dual-Band Array Topologies Based on Microstrip and Dipole Structures

**Figure 13:**
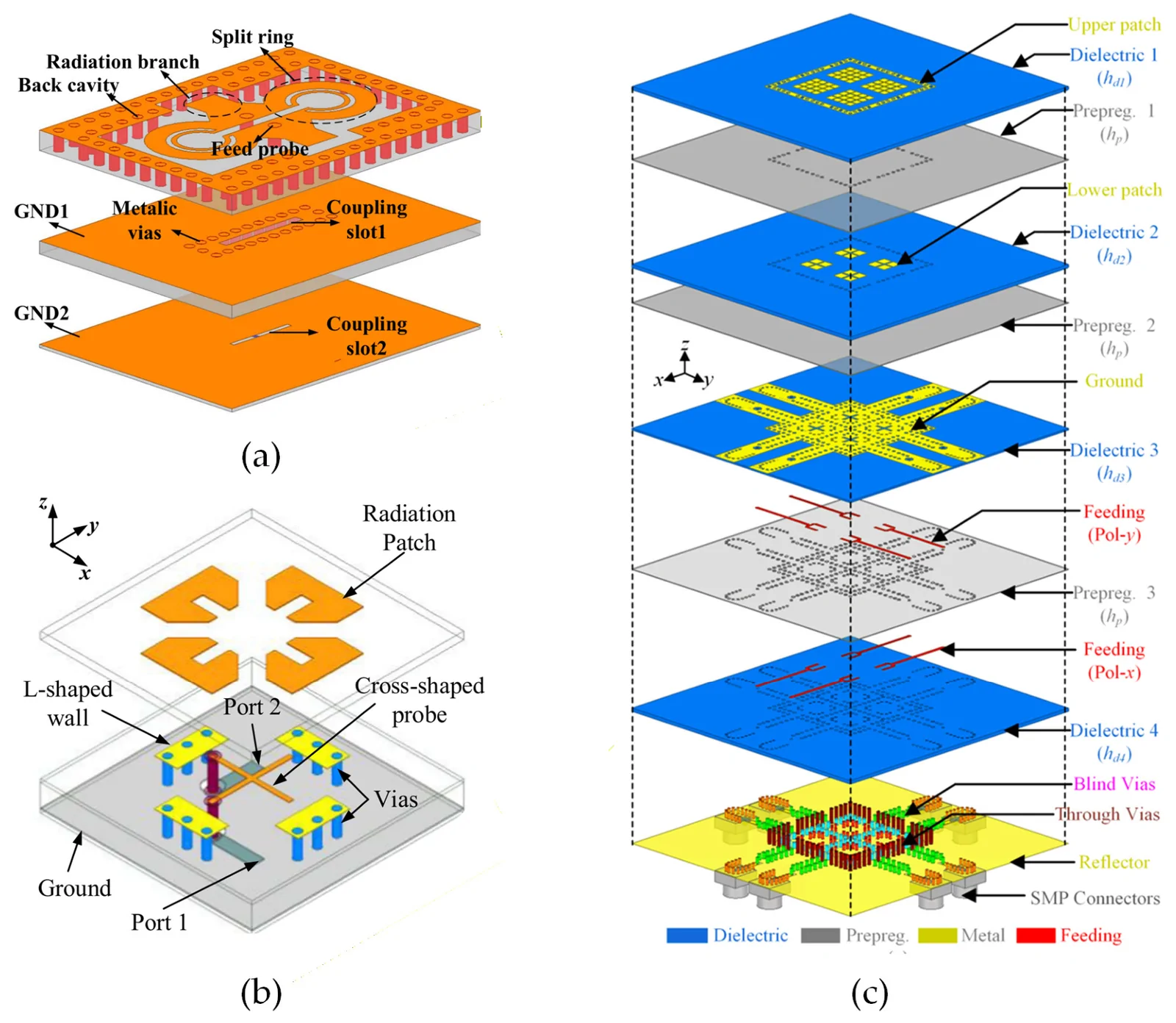
Representative dual-band millimeter-wave microstrip antenna configurations. (**a**) Microstrip dual-split-ring antenna [32]. (**b**) Modified rectangular patch loaded with shorted L-shaped strips for dual-polarized radiation [61]. (**c**) Double-layer gridded patches with metallic via fences for wide-angle beam scanning [62].

Microstrip patches and planar dipoles are widely used for dual-band millimeter-wave antennas owing to their ease of RF integration and flexible multiresonant characteristics [32,59,60,61,62]. Representative microstrip-based configurations are shown in Figure 13. In ref. [32], a dual-split-ring microstrip antenna array was proposed, where the inner and outer rings excite two resonant frequencies at 28 and 38 GHz, respectively. Furthermore, additional central branches are used to improve the radiation performance at the higher band. To support polarization diversity in millimeter-wave MIMO systems, dual-polarized designs have also been investigated. In ref. [61], a modified rectangular patch was loaded with shorted L-shaped strips. Dual-band dual-polarized radiation was achieved using a crossed microstrip feeds. In ref. [62], double-layer gridded patches and metallic via fences were introduced. This configuration broadens the impedance bandwidth of both bands and enables wide-angle beam scanning with a 2 × 2 array.

### 4.2. Dual-Band Millimeter-Wave Antennas Based on SIW Cavities

**Figure 14:**
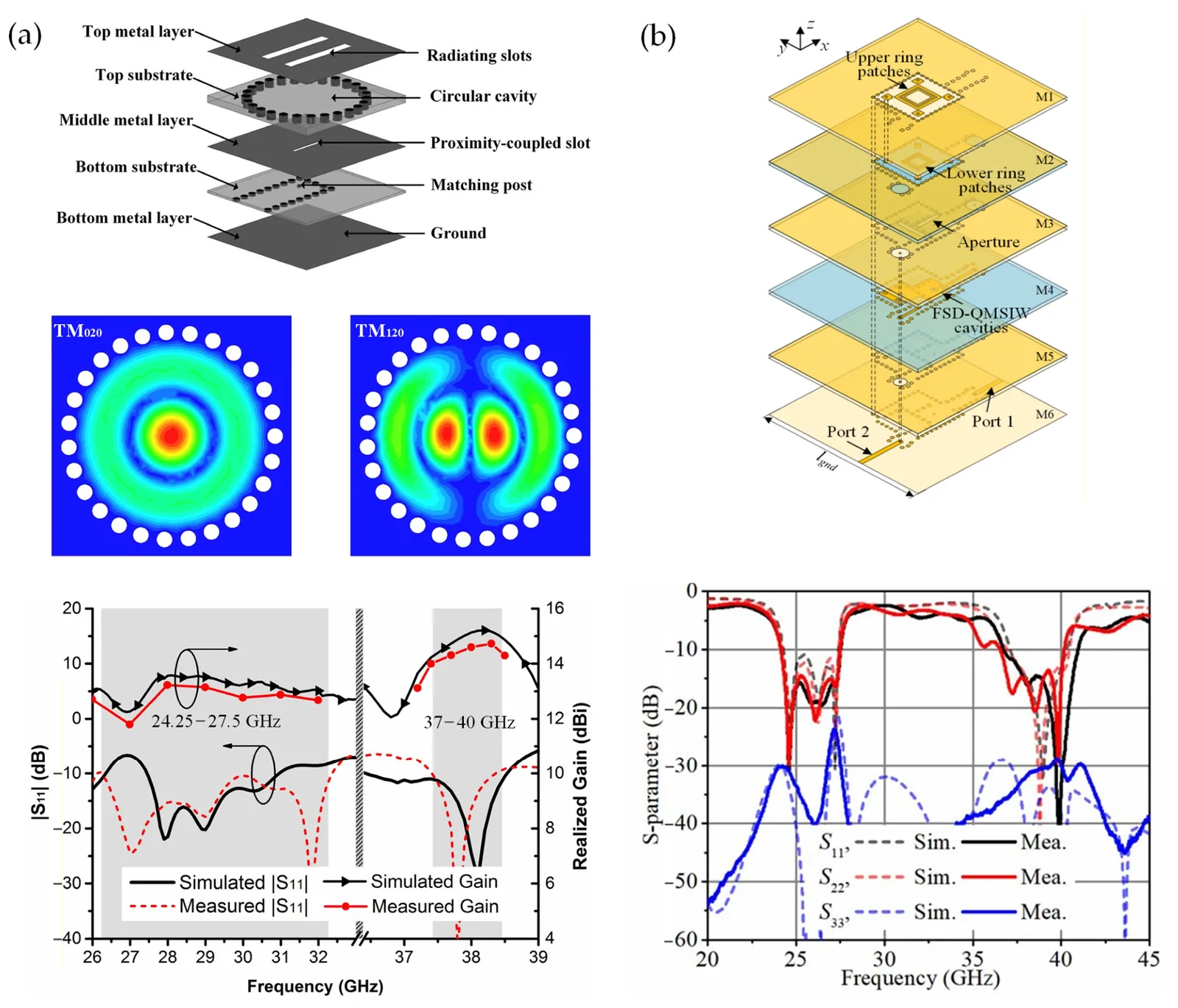
Representative architectures of millimeter-wave dual-band SIW antennas. (**a**) SIW cavity-backed slot array using TM_020_ and TM_120_ higher-order modes [33]. (**b**) Dual-polarized filtenna based on FSD-QMSIW cavities and stacked-ring radiators [63].

Conventional cavity-backed antennas are often bulky, and their multiple resonances can be difficult to control. To address these limitations, a low-profile dual-band SIW slot array was proposed, as illustrated in Figure 14a [33]. The TM_020_ and TM_120_ modes are excited through a proximity-coupled slot, which also suppresses the polarization-degenerate modes. The antenna achieves a profile of only 0.12λ, with peak gains of 13.2 and 14.6 dBi at 28 and 38 GHz, respectively. SIW cavities can also be coupled to other radiators to realize filtering responses. In ref. [63], dual-mode fully shielded quarter-mode substrate-integrated waveguide (FSD-QMSIW) cavities were coupled to stacked-ring radiators to form a dual-band dual-polarized filtenna. The coupled resonators generate an interband transmission null and additional radiation nulls, thereby improving out-of-band rejection.

### 4.3. Summary and Comparison

Table 3 summarizes representative dual-band millimeter-wave antennas and arrays. Microstrip-based approaches offer flexible dual-band and dual-polarized designs. In contrast, SIW-based antennas provide strong field confinement and high gain, while SIW filtennas further improve out-of-band rejection. These benefits are generally obtained at the cost of multilayer fabrication and more constrained modal tuning.

## 5. Sub-6 GHz and Millimeter-Wave Integration

Beyond dual-band millimeter-wave antenna design, the coexistence of Sub-6 GHz and millimeter-wave systems on a common smartphone platform has emerged as another important research topic. Sub-6 GHz and millimeter-wave antenna systems differ significantly in operating wavelength, radiation mechanism, and physical size. In compact smartphones, Sub-6 GHz antennas and millimeter-wave modules are often placed in close proximity. The resulting near-field coupling and capacitive loading make their co-integration particularly challenging. Therefore, the integration and coexistence of cross-band antennas have become important research topics in modern mobile terminal design.

### 5.1. Shared-Aperture and Shared-Surface Integration

**Figure 15:**
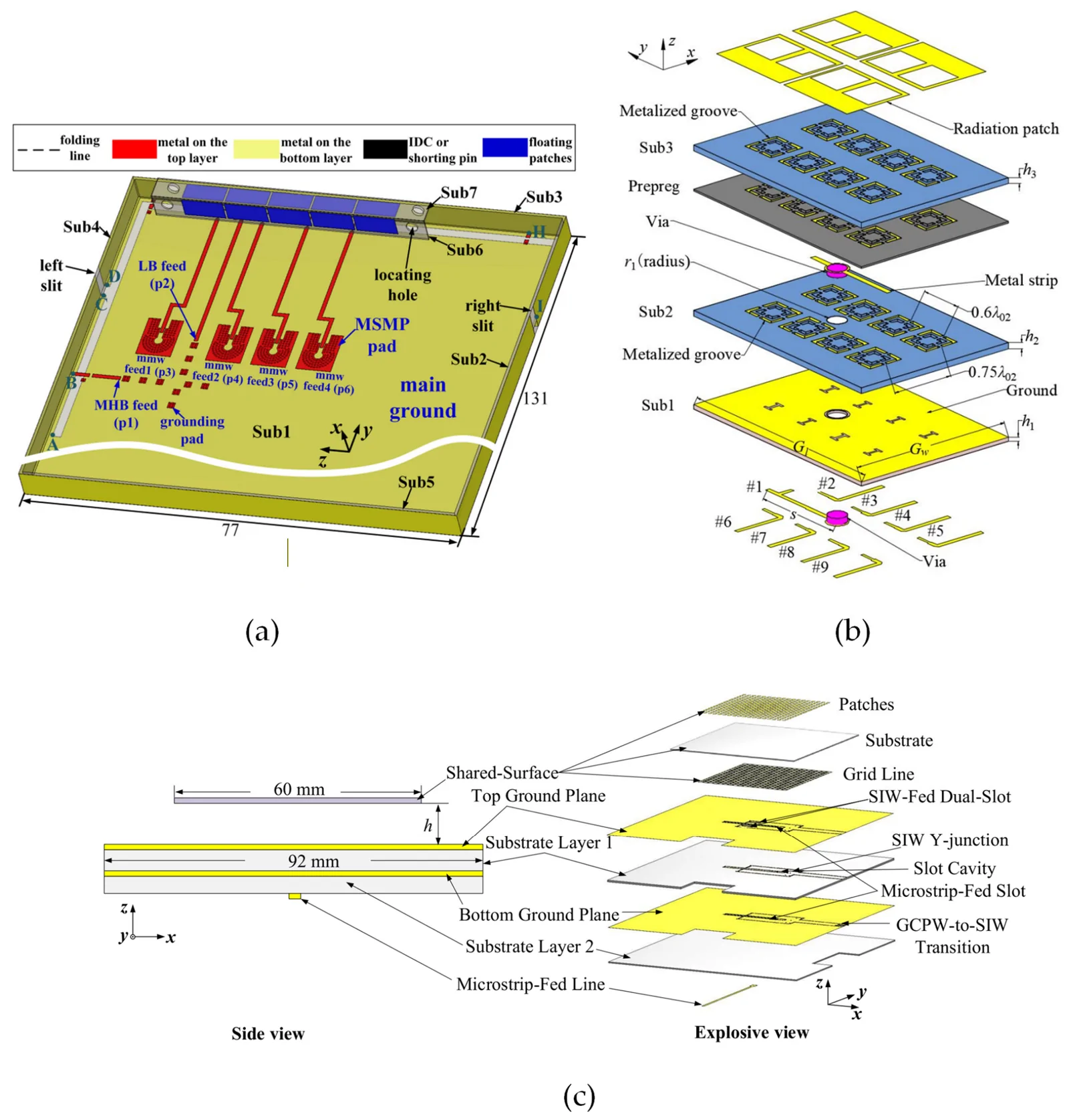
Representative Shared-aperture and shared-surface cross-band integration architectures. (**a**) 5G millimeter-wave array within the 4G LTE clearance area using an interdigital ground [64]. (**b**) Broadside shared-aperture 26 GHz array and 3.5 GHz perforated patch [65]. (**c**) Shared-surface combining an S-band metasurface and Ka-band partially reflective surface [66].

In order to improve space utilization in mobile terminals, shared-aperture integration has become a direct and effective approach [64,65,66]. Representative shared-aperture and shared-surface configurations are illustrated in Figure 15. In ref. [64], a millimeter-wave array was integrated into the available space of a 4G LTE antenna. An interdigital coupling structure was used as the ground plane for the millimeter-wave elements, while the low-frequency LTE antenna maintained its performance. In ref. [65], a 26 GHz array and a 3.5 GHz perforated patch antenna were integrated on the same substrate. Metallized grooves were introduced to suppress cross-band coupling in the broadside shared-aperture configuration. In ref. [66], a metasurface-based shared-surface scheme was introduced. By combining an S-band metasurface with a Ka-band partially reflective surface, the design enables the same physical surface to achieve good radiation performance at both low and high frequencies.

### 5.2. Structural Reuse and Mode Composite

**Figure 16:**
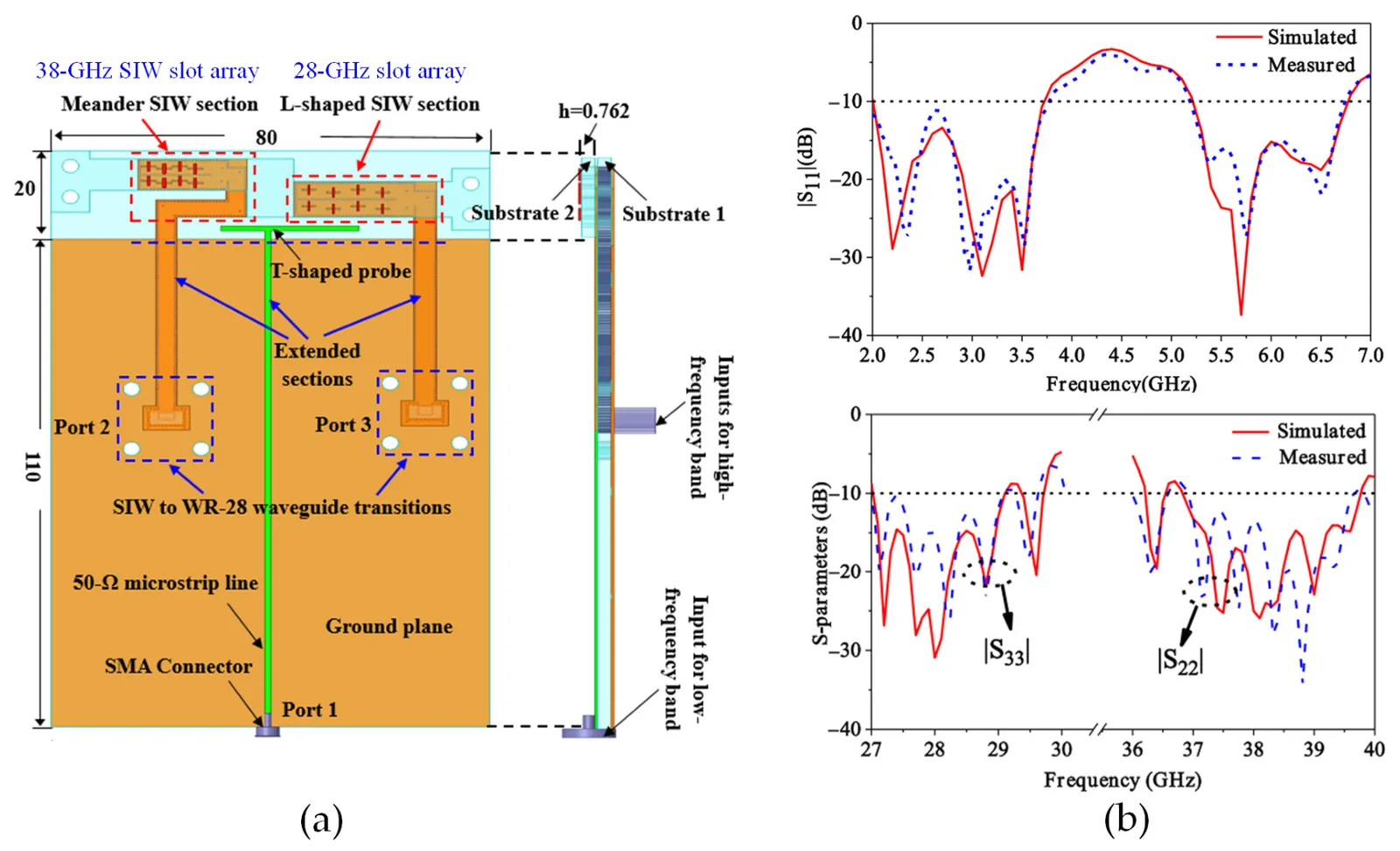
Hepta-band mode-composite antenna featuring internal SIW radiation (28/38 GHz) and outer surface reuse (Sub-6 GHz) [67]. (**a**) Antenna structure. (**b**) Simulated and measured S-parameters.

In addition to shared-aperture integration, mode-composite design enables multiband operation by reusing the same metallic structure to support different modes [67,68,69]. In ref. [67], cross-band operation was completed by combining both the waveguide modes and surface-current modes, as illustrated in Figure 16. The 28/38 GHz millimeter-wave signals propagate inside an SIW structure through the quasi-TE_10_ mode, while the outer surface of the SIW works as a wire antenna for Sub-6 GHz radiation. Thus, one metallic structure is able to support both millimeter-wave and low-frequency operation. Similarly, refs. [68,69] further explored structural reuse by employing slot-based designs and multi-mode array configurations.

### 5.3. Electromagnetic Transparency and Coexistence

**Figure 17:**
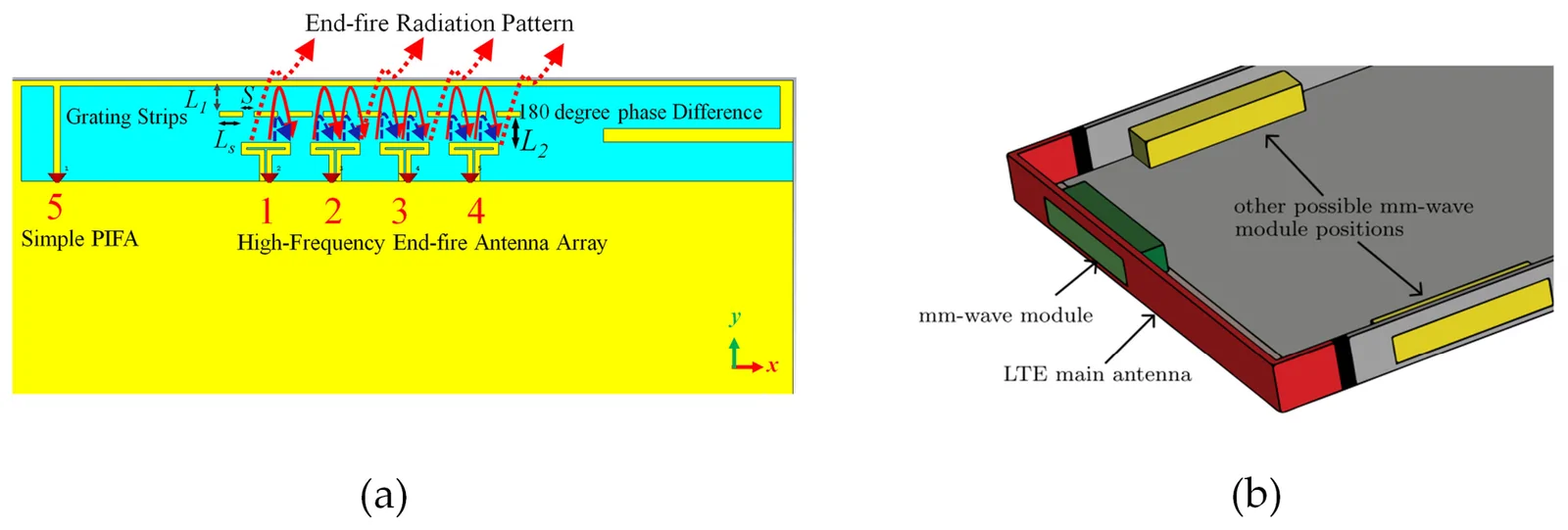
Coexisting Sub-6 GHz/millimeter-wave antenna architectures. (**a**) Electromagnetic-transparency integration using grating strips [70]. (**b**) Co-designed millimeter-wave/LTE antenna with a window structure [71].

In coexistence scenarios, near-field coupling and capacitive loading between the low- and high-frequency antennas greatly affect their respective performance. To address this issue, decoupling structures and band-stop elements have been widely used [22,70,71]. As illustrated in Figure 17a, ref. [70] employed an electromagnetic-transparency mechanism by inserting grating strips between a low-frequency PIFA and a millimeter-wave endfire array. Such grating strips reduce the influence of the PIFA on the millimeter-wave array, thereby enabling compact integration while ensuring relatively stable radiation patterns. A co-designed millimeter-wave/LTE antenna was reported in [71], as shown in Figure 17b. The millimeter-wave module was embedded in a window of the metal-rimmed LTE antenna. The co-designed structure enables simultaneous LTE and millimeter-wave operation with limited mutual interference.

### 5.4. Summary and Comparison

Table 4 summarizes representative integrated Sub-6 GHz and millimeter-wave antennas. Shared-aperture designs improve space utilization, whereas mode-composite schemes achieve deeper structural reuse at the cost of more involved cross-band modal control. Electromagnetic-transparency and co-designed approaches alleviate radiation blockage and extend angular coverage but introduce additional structures, matching networks, or switching circuits.

## 6. Emerging Technologies Toward B5G/6G Mobile Terminals

Although some of the technologies discussed below have already been investigated in current 5G systems, they are expected to play increasingly important roles in future 6G smartphones. In particular, integrated sensing and communications (ISAC) will require the coordinated integration of heterogeneous antenna modules across multiple frequency bands. As the number of supported bands and wireless functions continues to increase, smartphone antenna systems are evolving toward cross-band integration, AI-assisted design and evaluation, and operation at emerging FR3 and Sub-THz frequencies.

### 6.1. Cross-Band Hybrid Integration for ISAC

**Figure 18:**
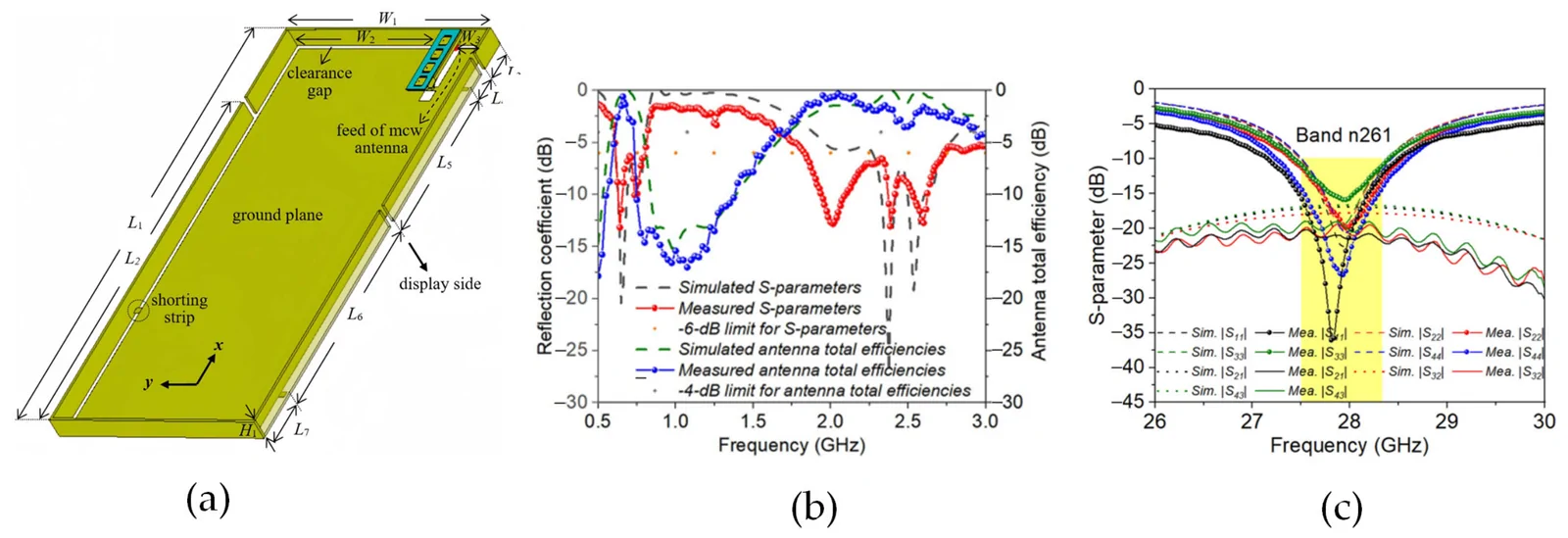
Hybrid integration of an FPC-based millimeter-wave module and a metal-frame Sub-6 GHz antenna [73]. (**a**) Antenna structure. (**b**) Simulated and measured $S_{11}$ and total efficiency of the Sub-6 GHz antenna. (**c**) S-parameters of the millimeter-wave antenna array.

ISAC-oriented mobile terminals may require antenna modules operating at different frequency bands for communication and sensing. One possible configuration combines a millimeter-wave sensing module with a Sub-6 GHz communication antenna [73]. In ref. [73], an FPC-based millimeter-wave AiP module operating in the n261 (27.5–28.35 GHz) band was integrated with a smartphone metal frame, as shown in Figure 18a. The metal frame serves as the Sub-6 GHz antenna, while the shielding case and hollow fixture of the millimeter-wave module are reused as parts of the low-frequency radiator. Joint tuning improves the lower-band impedance matching, as demonstrated by the $S_{11}$ results in Figure 18b. The measured millimeter-wave responses in Figure 18c confirm n261-band operation. This hybrid configuration improves space utilization and provides a potential platform for ISAC-enabled mobile terminals.

### 6.2. AI-Assisted Design and Evaluation of Multiband Smartphone Antennas

Artificial intelligence offers an effective means of reducing the computational cost of multiband smartphone antenna optimization and terminal-level evaluation. In ref. [72], machine learning was applied to accelerate the parameter optimization of a smartphone-sized MIMO platform covering the Sub-6 GHz bands at 2.4, 3.3, and 5 GHz and the millimeter-wave band of 23–29.5 GHz. In addition to antenna design, AI can support over-the-air performance assessment. In ref. [74], a deep learning framework predicted the EIRP, TRP, and SAR from near-field electromagnetic distributions under different antenna types, frequencies, and installation conditions. However, model generalization across different smartphone chassis, operating bands, user-hand scenarios, and manufacturing tolerances remains challenging.

### 6.3. Extending Smartphone Antennas Toward FR3 and Sub-THz

The upper-mid band, or FR3, is a promising candidate for 6G. It provides a balance between the coverage of Sub-6 GHz systems and the bandwidth available at millimeter-wave frequencies. In ref. [75], a low-profile 16-port MIMO array operating over 7.025–8.4 GHz was integrated into the back-cover region of a smartphone-sized platform. At higher frequencies, ref. [76] demonstrated a handset reference array operating at approximately 140 GHz on a phone-sized metallic chassis. These studies suggest a hierarchical architecture in which metal-frame antennas cover the lower bands, FR3 arrays support higher-order MIMO, and compact packaged arrays provide millimeter-wave and Sub-THz operation.

### 6.4. Open Challenges and Research Gaps

Despite recent progress, several research gaps remain in multiband antenna design for 6G smartphones. First, the increasing number of operating bands and wireless functions intensifies competition for antenna volume and aggravates cross-band interaction. Second, high-frequency arrays are sensitive to user blockage, assembly errors, and calibration drift, which may significantly reduce practical coverage. Finally, most studies still optimize antennas separately from the RF front end and packaging, while only a few demonstrate complete handset prototypes under realistic operating conditions. Future research should therefore focus on system-level co-design and consistent experimental evaluation.

## 7. Conclusions

This review has presented the main antenna technologies for multiband operation in modern smartphones. The reviewed topics are summarized as follows.

A. Single-antenna multiband techniques include multibranch radiators, multi-mode antennas, theory-of-characteristic-modes-based designs, and frequency-reconfigurable antennas. These approaches generate or control multiple resonances within a limited antenna region and form the basis of compact multiband smartphone antennas.

B. Multiband multi-antenna systems include filter-loaded and self-decoupled MIMO antennas, shared-branch architectures for 4G/5G coexistence, and circularly polarized antennas for terrestrial and satellite communications. These studies extend multiband design from individual radiators to coordinated antenna systems supporting multiple ports and communication functions.

C. Dual-band millimeter-wave antennas have been developed using planar and SIW-based structures. Their design has gradually extended from dual-resonant elements to array-level implementations with polarization and radiation-pattern control. These designs support beam-steering operation, while some SIW configurations further integrate filtering functions.

D. Sub-6 GHz and millimeter-wave antennas have been integrated through aperture sharing, structural reuse, and electromagnetic-transparency techniques. The reviewed studies focus on improving space utilization while maintaining the operation of both frequency ranges on a common smartphone platform.

For B5G and 6G smartphones, recent studies further indicate a transition toward cross-band hybrid integration for integrated sensing and communications, AI-assisted antenna design and evaluation, and operation at emerging FR3 and Sub-THz frequencies. Although several of these technologies remain at an early stage of handset implementation, they provide useful foundations for future multiband architectures. Further progress will require system-level co-design and handset-level validation under realistic operating conditions.

## Figures and Tables

**Figure 4:**
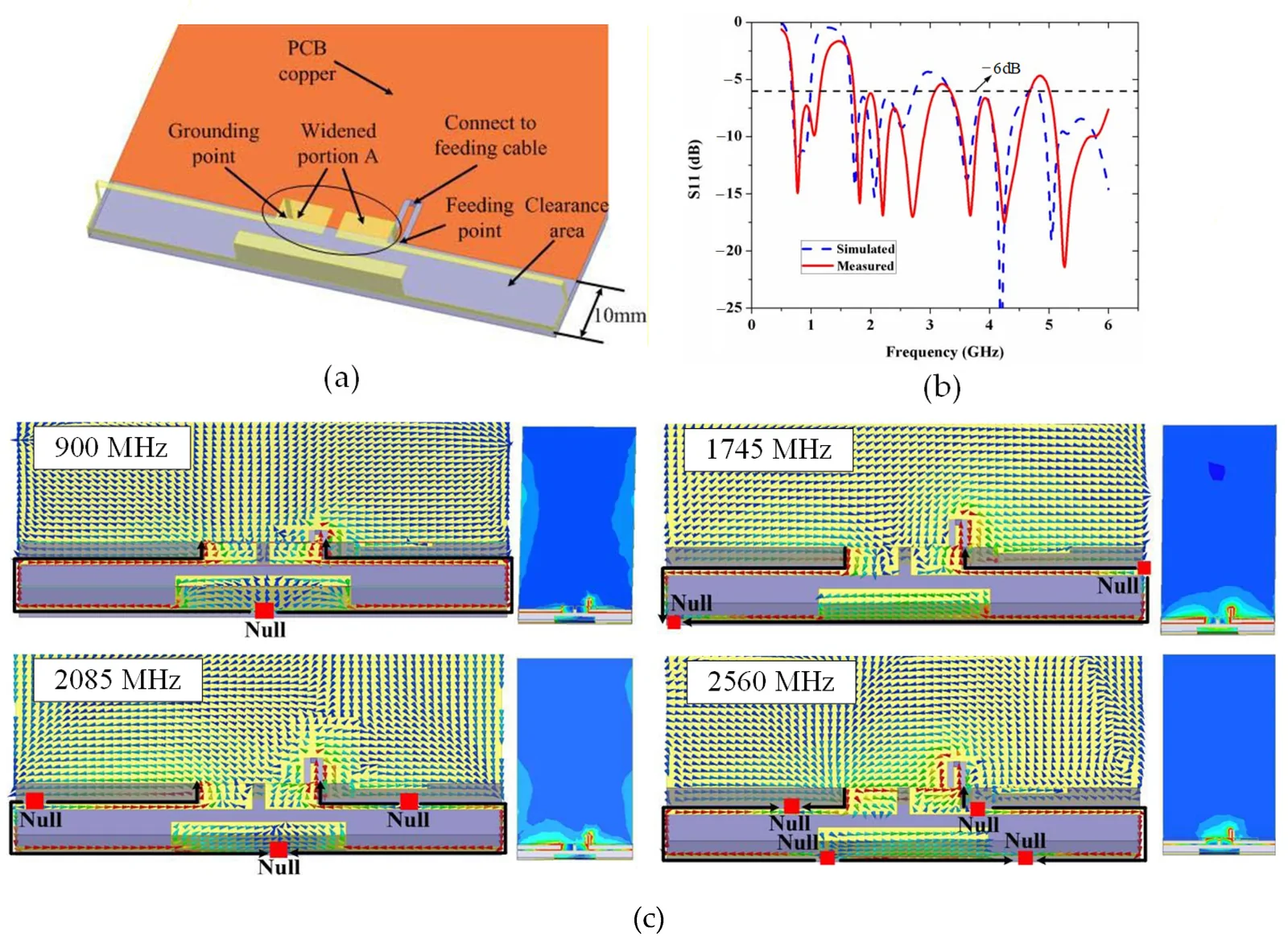
Multi-mode cooperation on a shared radiator [37]. (**a**) Geometry of a four-mode loop antenna. (**b**) Simulated and measured S11. (**c**) Vector surface current distributions and current density distributions.

**Table 1:** Comparison of representative single-antenna multiband techniques for smartphones.

Ref.	Category	Antenna Configuration	Freq. (GHz)	Main Technique	Advantage	Limitation
[6]	Multi-branch	PIFA–slot antenna	0.88–0.96, 1.71–2.17, 2.630–2.655	Parallel excitation of PIFA and slot radiators	Independent band control	Sensitivity to handset components
[37]	Multi-mode	Parasitic-loaded loop antenna	0.66–1.10, 1.71–3.02, 3.37–3.90, 5.15–5.85	Four loop modes and dual-mode parasitic loading	Broad coverage and user-interaction robustness	Complex mode tuning
[16]	TCM-based	Unbroken metal bezel	0.805–0.995, 1.34–2.87	TCM-guided bezel-mode excitation and mode merging	Preserved bezel integrity	Strong dependence on bezel modes
[46]	Reconfigurable	Folded loop–IFA	0.79–0.87, 1.490–2.225, 0.845–0.980, 2.240–2.565	PIN-controlled loop/IFA switching with a matching bridge	Compact hepta-band coverage	Switch loss
[47]	Reconfigurable	Metal-frame loop	0.698–0.960, 1.71–2.69	SP4T-controlled capacitive tuning of loop modes	Full LTE coverage in a compact frame	Switch loss

**Table 2:** Comparison of representative multiband multi-antenna systems for smartphones.

Ref.	Category	Antenna Configuration	Freq. (GHz)	Main Technique	Advantage	Limitation
[26]	Multiband 5G MIMO	T-monopole and T-slot antenna pair	3.40–3.60, 4.80–4.90	Polarization-orthogonal decoupling	Dual-band operation and cross-band decoupling	Finite-Q loss and out-of-band efficiency nulls
[53]	Multiband 5G MIMO	Asymmetric antenna pair	(Ant1)0.88–0.96, 2.50–2.69, 3.40–3.60; (Ant2)2.50–2.69, 3.40–3.60, 4.80–4.90	Self-multipath decoupling	Tri-band self-decoupling without extra structures	Asymmetric band coverage
[28]	4G/5G coexistence	Shared-branch metal-bezel	0.698–1.010, 1.62–2.81, 3.25–4.23, 4.39–5.01	Capacitor-loaded branch reuse	Multiband coverage with small clearance	Complex branch and matching network tuning
[29]	Satellite-cellular integration	Folded loop and parasitic branch	(CEL)0.704–0.976, 1.66–2.78; (n256)1.92–2.05, 2.160–2.225	Orthogonal-mode excitation	Dual-band endfire CP with cellular coexistence	Strict modal tuning and limited CP gain
[58]	Reconfigurable satellite CP	Metal bezel and parasitic patch	1.575–1.655, 2.055–2.155, 3.475–3.530	Switch-controlled HP/VP mode tuning	Single-feed tri-band endfire CP	Non-simultaneous operation

**Table 3:** Comparison of representative dual-band millimeter-wave antennas and arrays.

Ref.	Category	Antenna Configuration	Freq. (GHz)	Main Technique	Advantage	Limitation
[32]	Microstrip array	1 × 4 split-ring array	26.0–29.5, 36.7–38.7	Dual split-ring resonances with parasitic branches	Single-chain dual-band operation with high gain	Narrow upper band
[62]	Dual-polarized microstrip array	2 × 2 chamfered-patch array	23.5–30.8, 36.7–47.3	Patch–strip multi-mode coupling	Compact wideband dual-polarized coverage	Moderate port isolation
[63]	Dual-polarized microstrip array	2 × 2 gridded-patch array	23.3–31.7, 42.5–46.5	TM_10_/antiphase-TM_20_ mode excitation	Wideband dual polarization with beam scanning	Reduced high-band scan range
[33]	Single-polarized SIW array	SIW slot array	26.3–32.3, 37.4–38.4	TM_020_/TM_120_ cavity-mode excitation	Low profile and high gain	Narrow upper band
[64]	Dual-polarized SIW filtenna array	FSD-QMSIW filtenna array	24.25–27.50, 37.0–40.0	Dual-mode FSD-QMSIW filtering with stacked-ring radiation	Dual-polarized coverage with strong out-of-band rejection	High fabrication complexity

**Table 4:** Comparison of representative Sub-6 GHz and millimeter-wave integrated smartphone antennas.

Ref.	Category	Antenna Configuration	Freq. (GHz)	Main Technique	Advantage	Limitation
[65]	Shared-aperture integration	Metal-bezel antenna with slot array	0.70–0.98, 1.70–2.92, 25.1–27.8	IDC-ground aperture sharing and floating patch loading	display-direction beam steering	High fabrication complexity
[66]	Shared-aperture integration	Perforated-patch/SIDRA array	3.30–3.71, 24.75–27.5	Perforated-patch/SIDRA aperture sharing with groove isolation	Broadside beam steering and low cross-band coupling	High fabrication complexity
[68]	Mode-composite integration	SIW structure with slot arrays	2.00–3.77, 5.15–6.75, 27.9–29.0, 36.9–39.6	Internal SIW modes and outer-surface wire modes	Hepta-band coverage with structural reuse	Low gain in the Sub-6 GHz bands
[71]	Electromagnetic-transparency integration	PIFA with folded-dipole array	0.74–0.96, 1.7–2.2, 22.0–31.0	Anti-reflective grating-strip transparency	Wideband endfire beam scanning with compact coexistence	Quarter-wave spacing constraint
[72]	Shared-aperture co-design	CCE with parasitic-patch arrays	0.75–1.13, 1.70–5.30, 27.50–28.35	CCE-based chassis-mode excitation and PIN-switched parasitic beam steering	Wide Sub-6-GHz coverage and 360° mm-wave coverage	Discrete two-state beam steering

Note: IDC: interdigital coupling; SIDRA: substrate-integrated dielectric resonator antenna; CCE: capacitive coupling element.

## Data Availability

The original contributions presented in the study are included in the article; further inquiries can be directed to the corresponding author.
